# Fox River Valley Med. Assoc.

**Published:** 1867

**Authors:** C. Cushing

**Affiliations:** Sec’y.


### PROCEEDINGS OF MEDICAL SOCIETIES.

### FOX RIVER VALLEY MEDICAL ASSOCIATION.

The Association met at Turner Junction, DuPage Co., Ill., on the 1st of April, and was called to order by the President.

After the ordinary preliminary business, Drs. Hard, LeBaron, Winchester, and D. W. Young were elected delegates to the State Medical Association; and Drs. Hawley, Hard, Dean, and Hagar were elected delegates to the American Medical Association.

Dr. Hard delivered an address upon the influence of evil habits in the production of disease, with special reference to the use of tobacco. The address elicited a lively discussion, which was participated in by all the members present.

Drs. Hawley, Winchester, and Cushing, each reported an interesting case of injury of the head, one of which proved fatal, one recovered, and one still under treatment.

Dr. Hard reported a case of melanotic tumor, which had been twice removed within three months.

A discussion then followed upon obstetric practice, by Drs. Hawley, LeBaron, and Woodworth.

The Secretary, Dr. Cushing, being about to remove to California, tendered his resignation, and Dr. Hard was elected to fill the vacancy. The Association passed, unanimously, resolutions of respect for the retiring Secretary.

On motion of Dr. Eddy, the Association adjourned to meet at Turner Junction, on the first Monday of July next.

C. CUSHING, M.D., *Sec'y.*

